# An unusual presentation of dermatitis in a Yao syndrome patient

**DOI:** 10.1016/j.jdcr.2025.11.014

**Published:** 2025-11-19

**Authors:** Saira Alvi, Liam Bloebaum, Jack Hulse, Allison Swanson, Brittany Oliver, Anand Rajpara

**Affiliations:** a Department of Dermatology, University of Missouri-Kansas City School of Medicine, Kansas City, Missouri; b A.T Still University, Kirksville College of Osteopathic Medicine, Kirksville, Missouri

**Keywords:** autoinflammatory disease, eosinophilic dermatosis, NOD2 mutation, Wells syndrome, Yao syndrome

## Introduction

Yao syndrome (YAOS), first described in 2011 by Yao et al, is a novel autoinflammatory disease associated with nucleotide oligomerization domain 2 (NOD2) gene mutations.^1^ NOD2 is implicated in inflammatory regulation, apoptosis, and cytokine processing and is associated with pediatric Blau syndrome and Crohn's disease.^2^^,^^3^

Patients commonly present between the ages of 20 and 50 with minimally pruritic, erythematous plaques, patches, papules, and linear scratch-like rashes on the face, trunk, and extremities. The rash usually lasts several days.^1^^,^^4^^,^^5^ Other symptoms include periodic fever, polyarthralgias, periorbital and distal extremity edema, and gastrointestinal symptoms. There is a 2:1 female predominance.^2^^,^^4^^,^^5^ Histology usually shows spongiotic dermatitis; however, other histological variants have been described.^4^^,^^5^

Due to the episodic presentation of the disease, courses of oral glucocorticoids are often used, with sulfasalazine employed in patients with more frequent attacks. More than 50% of patients have been seen to respond to daily sulfasalazine. In patients who failed or have contraindications to those options, IL-1/IL-6 inhibitors can be utilized.^3^^,^^6^^,^^7^

Wells syndrome, or eosinophilic cellulitis, is a rare inflammatory dermatosis characterized most commonly by recurrent, erythematous, edematous plaques. Patients may less commonly present with vesicles, bullae, or pustules and associated fever, malaise, and arthralgias. Histologically, it features a dermal eosinophilic infiltrate and distinctive flame figures formed by eosinophil granule deposition on collagen bundles.^8^ The pathogenesis remains incompletely understood but appears to involve a dysregulated eosinophil-mediated immune response triggered by infections, medications, arthropod bites, or systemic diseases. We report the case of a man with YAOS presenting with a rash characteristic of Wells syndrome refractory to corticosteroids and conventional disease-modifying antirheumatic drugs (DMARDs). We also review the literature, highlighting differences in typical patient presentations that demonstrate the variable cutaneous manifestations of this disease.

## Case report

We report the case of a 70-year-old man with common variable immunodeficiency, seronegative rheumatoid arthritis, coronary artery disease, hypertension, iron deficiency anemia, gastroesophageal reflux disease, hyperlipidemia, obstructive sleep apnea, type 2 diabetes mellitus, asthma, and nephrolithiasis. He carried a previously established diagnosis of YAOS.

**Fig 1:**
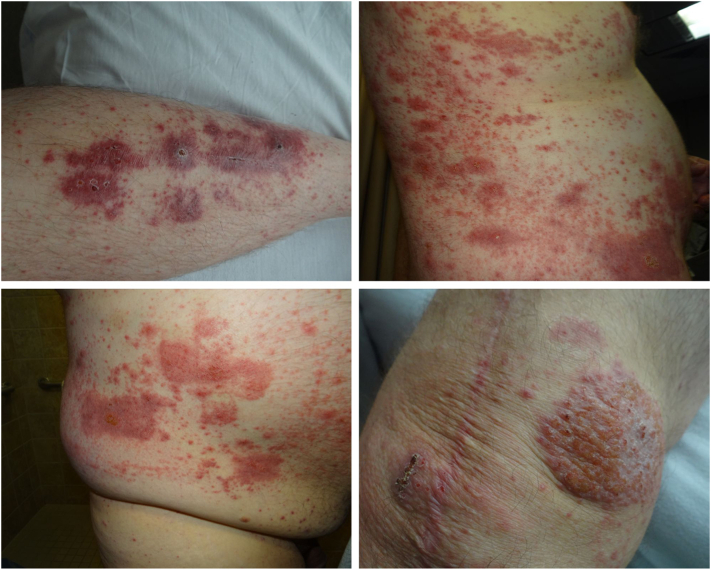
YAOS clinical presentation of the patient's initial eruption in 2013 demonstrating widespread erythematous macules, papules, and plaques with overlying scale, excoriations, and crusting. Lesions are distributed across the extremities and trunk, including well-demarcated plaques with lichenification on the knee, coalescing plaques on the abdomen and flank, and grouped plaques with central ulceration on the lower leg. *YAOS*, Yao syndrome.

His symptoms started 12 years prior, beginning with a recurrent vesiculopapular eruption at the site of a total knee replacement, which spread to his extremities, trunk, and head (Fig 1). The eruption cycled every 3-4 weeks and was associated with intense pruritus, crusting, and incomplete response to corticosteroids. Over several years, he experienced episodic fever, diffuse rash, arthralgias, and systemic inflammation. Hospitalizations for presumed sepsis were frequent, though cultures consistently returned negative. Multidisciplinary evaluation yielded a differential including contact dermatitis (in the setting of a known metal allergy), impetigo, drug eruption, scabies, and autoimmune conditions.

Initial autoimmune and infectious workups were unremarkable. He reported frequent infections since his 30s and in 2014 was diagnosed with common variable immunodeficiency (IgG 617 mg/dL, IgM 10 mg/dL). He began subcutaneous immunoglobulin (Hizentra) therapy. He also developed arthralgias and swelling attributed to seronegative rheumatoid arthritis, treated with methotrexate, leflunomide, and hydroxychloroquine with symptomatic improvement except for his rash.

In 2015, he developed worsening constitutional symptoms, including fatigue, fevers, malaise, and abdominal pain. Laboratory workup revealed lymphopenia (CD3 308, CD4 287, CD8 27), markedly elevated CRP >180 mg/L, ESR >100 mm/hr, and ferritin >1000 ng/mL. Computed tomography scan demonstrated a “misty mesentery”, and laparoscopy revealed fat necrosis with septal fibrosis without evidence of malignancy or vasculitis. HLH was ruled out given that diagnostic criteria were not met as the patient exhibited only fever and elevated ferritin levels (>500 ng/mL). Trials of antibiotics were ineffective. High-dose oral corticosteroids temporarily resolved symptoms, but recurrent episodes continued.

In early 2016, he was referred to Mayo Clinic for further evaluation. Additional immunologic and genetic testing including IL-1β, TNF-α, IL-6, Whipple’s PCR, IgG4, PR3, MPO, and TNFRSF1A was unremarkable. A variant of uncertain significance in the NOD2 gene was identified. Clinical course, genetic findings, and exclusion of other etiologies led to a diagnosis of YAOS. He was started on sulfasalazine, without improvement, then switched to abatacept which improved his rheumatologic symptoms.

**Fig 2:**
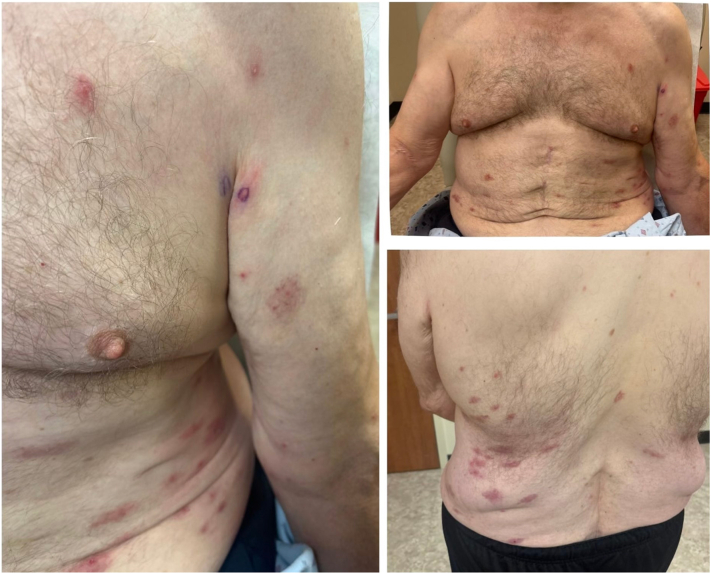
YAOS clinical examination following 3 days of oral and topical corticosteroids at most recent clinical visit in 2025 showing scattered erythematous papules and plaques with postinflammatory hyperpigmentation involving the upper trunk, chest, abdomen, and proximal upper extremities. Some lesions exhibit central clearing or crusting, while others are violaceous or excoriated. The eruption appears in partial resolution, with evidence of both active and resolving inflammatory lesions. *YAOS*, Yao syndrome.

**Fig 3:**
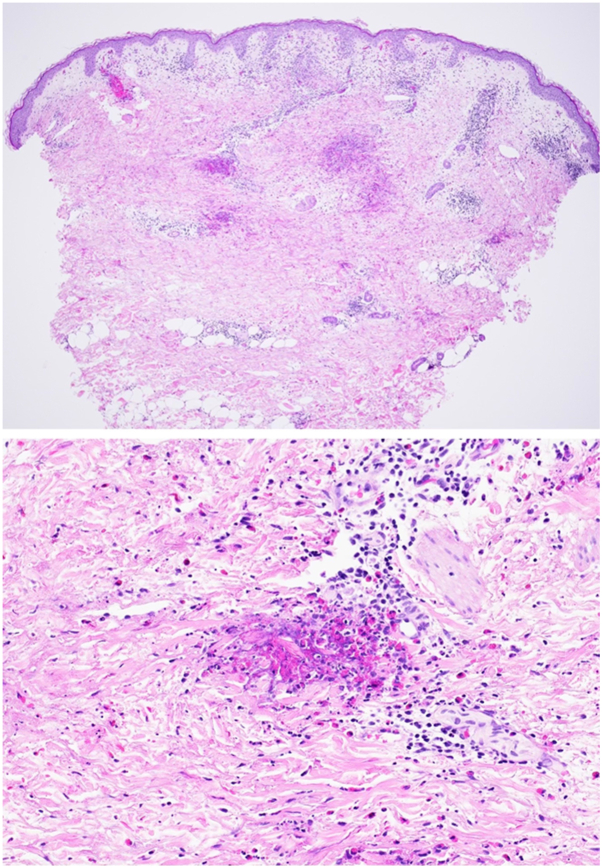
Punch biopsy taken from left upper arm showing YAOS histology demonstrates a collection of eosinophilic material deposited on collagen fibers and surrounded by eosinophils, otherwise known as “flame figures.” (hematoxylin-eosin, 200× original magnification). *YAOS*, Yao syndrome.

Most recently, he presented to our dermatology clinic with an abrupt-onset, 3-week history of a severely pruritic rash. He trialed a 3-day course of oral prednisone (40 mg daily) and topical betamethasone with only partial relief in pruritus. A prior dermatologist attributed the eruption to arthropod bites. Current medications included abatacept, pantoprazole, ropinirole, rosuvastatin, metaproterenol, semaglutide, and venlafaxine. He denied recent infections, new medications, insect exposure, or animal contact. He also denied oral or mucosal lesions, current gastrointestinal symptoms, or current rheumatologic symptoms. Laboratory data, including basic metabolic panel, comprehensive metabolic panel, and peripheral eosinophil count, were not obtained. Physical examination revealed a widespread erythematous maculopapular eruption involving the bilateral arms, legs, and trunk, with evolution into urticarial plaques and nodules (Fig 2). Lesions demonstrated surrounding erythema, edema, excoriations, and central ulceration. A 4 mm punch biopsy was performed. He was prescribed clobetasol 0.05% ointment twice daily (2 weeks on, 2 weeks off) and continued a prednisone taper (30 mg × 3 days, 20 mg × 3 days, 10 mg × 3 days). Histopathologic evaluation revealed eosinophilic material coating collagen fibers surrounded by eosinophils, otherwise called “flame figures” (Fig 3). Upon follow up to the dermatology clinic 1 month after our initial encounter, the patient had continuing moderate-to-severe erythematous plaques and pruritus involving the anterior and posterior trunk and proximal upper extremities. He stated that his symptoms improved on the steroid taper but returned after finishing the medication. The patient was started on dupilumab 300 mg subcutaneously every 2 weeks. After 1 month of dupilumab therapy, the patient reports no significant flares and denies any pruritus.

## Discussion

This case diverges from the classic presentation of YAOS in several ways. The typical YAOS rash consists of erythematous, edematous patches with minimal pruritus.^4^ In contrast, our patient developed intensely pruritic eruptions that were initially vesiculopapular but progressed to maculopapular with ulcerations, nodules, and plaques. Histopathologic findings were more characteristic of Wells syndrome.^4^^,^^5^^,^^8^^,^^9^ These differences suggest our patient may in fact have Wells syndrome superimposed on a known diagnosis of YAOS. Due to its rarity, it is also possible this could be a phenotypic expansion of YAOS with clinical and histological characteristics resembling Wells syndrome (Table I). Additionally, the fact that our patient presents with findings characteristic of 2 separate rare diseases makes this case particularly noteworthy and complex.

**Table I:** Comparison of clinical and histopathologic features between Yao syndrome and Wells syndrome

	YAOS	Wells syndrome
Etiology	Autoinflammatory disorder linked to NOD2 gene variant^1^^,^^4^^,^^5^	Idiopathic or hypersensitivity reaction
Onset	Often middle-aged adults	Any age
Clinical presentation	Recurrent fevers, urticarial or erythematous plaques, arthralgias, abdominal pain^1^^,^^4^^,^^5^	Recurrent itchy, burning erythematous plaques^9^
Systemic symptoms	Common	Rare; usually limited to skin
Lesion distribution	Nonspecific; trunk and extremities	Often localized; trunk and extremities^9^
Peripheral eosinophilia	Mild to absent	Common; about half of cases
Histopathology	Nonspecific dermal perivascular lymphocytic infiltrate; spongiotic dermatitis^1^^,^^4^^,^^5^	Dermal eosinophilic infiltrate; flame figures^9^
Vasculitis	Absent or mild	Absent
Key diagnostic features	Recurrent fevers, dermatitis, arthralgias, and abdominal pain; NOD2 variant for confirmation^1^^,^^4^^,^^5^	Recurrent eosinophilic plaques; flame figures on biopsy^9^

*NOD2*, Nucleotide oligomerization domain 2; *YAOS*, Yao syndrome.

The diagnostic process was complex. Rash morphology, intense pruritus, episodic distribution, and histology initially prompted consideration of eczematous dermatitis, drug eruption, arthropod bites, and Wells syndrome. Persistence of symptoms despite appropriate treatment, along with identification of a NOD2 variant, ultimately supported the diagnosis prior to the most recent presentation. This case highlights the importance of histopathologic evaluation and maintaining a broad differential diagnosis for recurrent, pruritic eruptions with systemic symptoms, even when histology is not classic.

Management of YAOS remains challenging. While 36% to 75% of patients respond to oral corticosteroids alone, others benefit from DMARDs such as methotrexate or sulfasalazine.^10^ In refractory disease, biologic agents targeting IL-1 or IL-6 have been used successfully.^6^^,^^7^ Our patient’s inadequate response to corticosteroids and DMARDs suggests earlier consideration of biologic therapy may improve quality of life and reduce corticosteroid exposure.

Corticosteroids remain first-line treatment for Wells Syndrome in most cases. In recent years, concern for relapse or adverse drug effects has led to an increasing amount of literature on possible use of steroid-sparing therapies. In the past decade, emerging case reports on the efficacy of biologics, such as dupilumab targeting the IL-4/IL-13 pathway and Janus kinase inhibitors such as topical ruxolitinib and oral abrocitinib have been shown to be useful in patients with steroid-resistant cases or in patients who are unable to utilize corticosteroids.^9^

This case represents a diagnostic challenge, with lesions and histology suggestive of Wells syndrome and a patient with prior known YAOS. The potential for YAOS to overlap with other eosinophilic dermatoses should be recognized, as misclassification can delay diagnosis and proper treatment. Awareness of this unusual presentation can support timely escalation to biologic therapy when first-line treatments such as corticosteroids fail. Further research into NOD-2 associated autoinflammatory diseases can help us to better understand dermatologic presentations of such conditions and help us to better manage patients with characteristic clinical, histologic, and genetic features.

## Conflicts of interest

None disclosed.
